# Repetitive transcranial magnetic stimulation improves both hearing function and tinnitus perception in sudden sensorineural hearing loss patients

**DOI:** 10.1038/srep14796

**Published:** 2015-10-14

**Authors:** Dai Zhang, Yuewen Ma

**Affiliations:** 1 Department of Rehabilitation Medicine, The First Affiliated Hospital, China Medical University, Shenyang, China.

## Abstract

The occurrence of sudden sensorineural hearing loss (SSHL) affects not only cochlear activity but also neural activity in the central auditory system. Repetitive transcranial magnetic stimulation (rTMS) above the auditory cortex has been reported to improve auditory processing and to reduce the perception of tinnitus, which results from network dysfunction involving both auditory and non-auditory brain regions. SSHL patients who were refractory to standard corticosteroid therapy (SCT) and hyperbaric oxygen (HBO) therapy received 20 sessions of 1 Hz rTMS to the temporoparietal junction ipsilateral to the symptomatic ear (rTMS group). RTMS therapy administered in addition to SCT and HBO therapy resulted in significantly greater recovery of hearing function and improvement of tinnitus perception compared SCT and HBO therapy without rTMS therapy. Additionally, the single photon emission computed tomography (SPECT) measurements obtained in a subgroup of patients suggested that the rTMS therapy could have alleviated the decrease in regional cerebral brain flow (rCBF) in SSHL patients. RTMS appears to be an effective, practical, and safe treatment strategy for SSHL.

Sudden sensorineural hearing loss (SSHL) is typically defined as >30 dB sensorineural hearing loss at 3 contiguous frequencies within an interval of <3 days. SSHL affects from 5 to 160 cases per 100,000 people per year^1^,^2^. Approximately 50% of SSHL cases may spontaneously recover^3^. The causes of most cases of SSHL cannot be identified and are considered to be idiopathic. The most common hypotheses regarding the causes of idiopathic SSHL (ISSHL) are circulatory disturbance to the end artery of the cochlea and viral infection. Based on these hypotheses, corticosteroids are provided as an initial therapy for ISSHL, and hyperbaric oxygen (HBO) therapy is implemented as an adjunctive treatment for ISSHL^1^. However, a recent meta-analysis of various medical treatments, including corticosteroids, showed that medical therapy produced a slight but not statistically significant improvement in audiograms compared with placebo^4^. Additionally, a recent randomized triple-blind placebo-controlled clinical trial demonstrated that corticosteroids administered at a customary dosage did not influence hearing recovery^5^. Therefore, new strategies to treat ISSHL are needed.

Recently, strategies to treat ISSHL have been proposed that are based on the reorganization of the auditory cortex after the occurrence of ISSHL^6^. In ISSHL patients, magnetoencephalography (MEG)^7^,^8^ and functional magnetic resonance imaging (fMRI)^9^ studies demonstrated that reorganization of the auditory cortex developed within a few days after the onset of hearing loss. This reorganization is a relatively rapid plastic event that begins within hours after cochlear trauma in an animal model^10^. These effects coincide with a range of central nervous system (CNS) changes, including the reorganization of frequency representation, alterations in the pattern of spontaneous activity and altered expression of excitatory and inhibitory neurotransmitters. Moreover, damage to the cochlea is often accompanied by acoustic disorders, such as hyperacousis and tinnitus, suggesting that one or more of these neuronal changes may be involved in this disorder, although its underlying mechanisms remain unknown. Moreover, the degree of cortical reorganization during the acute SSHL phase negatively correlated with the rate of recovery from hearing loss^11^. Therefore, it has been proposed that the prevention of maladaptive cortical reorganization associated with SSHL may represent a promising therapeutic strategy.

In the last decade, repetitive transcranial magnetic stimulation (rTMS) over the temporal or temporoparietal cortex has been investigated for the treatment of tinnitus, which appears to originate from maladaptive cortical reorganization. Tinnitus is associated with neural changes in both auditory (increase in the spontaneous stochastic firing rate, hyperactivity, alterations of the tonotopic map) and non-auditory brain areas^12^. Based on these findings, rTMS has been proposed as an innovative therapeutic strategy for tinnitus. Functional imaging studies have shown that individuals who experience tinnitus display increased activity in the auditory cortex^13^,^14^. Applying low-frequency TMS (e.g., 1 Hz), which is purported to reduce cortical activity^15^, to the auditory or associated cortex may reduce patients’ perceived severity or volume of tinnitus. Studies of the use of rTMS for chronic tinnitus showed that the severity of tinnitus was reduced by rTMS^16^,^17^, although some similar rTMS therapy studies showed no significant improvement in tinnitus severity^18^,^19^. Several additional trials were ongoing at the time of this study. In addition, the effect of 1 Hz rTMS applied to the auditory cortex (Heschl’s gyrus or temporoparietal area) on auditory processing has been studied, and the results demonstrated improved auditory processing in both normal hearing subjects and tinnitus patients following rTMS therapy^20^,^21^,^22^. Based on these data, we hypothesized that rTMS disrupts the process of cortical reorganization after SSHL thereby altering outcomes.

In this study, rTMS was performed for the first time as a treatment approach on ISSHL patients with incomplete hearing recovery. The therapeutic impact of 4 weeks of low-frequency (1 Hz) rTMS on these patients was investigated. The treatment outcomes were evaluated by comparing the pure tone audiograms of two groups of ISSHL patients: the control group (N = 20), which received standard corticosteroid therapy (SCT) and HBO therapy, and the target group (N = 34), which received rTMS therapy in addition to SCT and HBO therapy. Tinnitus volume and severity were analyzed for those patients exhibiting tinnitus symptoms. Moreover, via single-photon emission computed tomography (SPECT), we measured regional cerebral blood flow (rCBF) in a subgroup (N = 5) of patients in the target group who agreed to participate in SPECT measurement and for whom it was possible to arrange these measurements before rTMS therapy was initiated. The goal of these SPECT measurements was to investigate the degree of maladaptive cortical activity before and after rTMS therapy. It was hypothesized that in light of the observed improvements in auditory processing reported in tinnitus patients and healthy participants post-rTMS, individuals with short-term ISSHL would exhibit better hearing outcomes after the application of rTMS.

## Results

A total of 54 patients with ISSHL refractory to primary treatment were enrolled in this study. The rTMS group (n = 34) and the control group (n = 20) were classified according to whether or not the patients elected to receive rTMS therapy. The patients in each of the two groups did not differ regarding age (rTMS group: 45.45 ± 14.73 years; control group: 48.23 ± 15.24 years), the interval between the onset of hearing loss and initial treatment (rTMS group: 6.5 ± 3.2 days; control group: 7.25 ± 4.9 days), or the pure-tone average (PTA) (rTMS group: 65.16 ± 15.23 dB HL; control group: 68.95 ± 13.13 dB HL), which may affect hearing outcomes. The interval between the onset of hearing loss and rTMS therapy in the rTMS group was 44.8 ± 12.2 days. Before treatment, at all frequencies in the affected ears, the hearing thresholds were similar between the groups (mean hearing level (dB) ±standard deviation in the rTMS group: 0.25 kHz 53.9 ± 17.3, 0.5 kHz 68.0 ± 12.0, 1 kHz 70.7 ± 13.3, 2 kHz 71.6 ± 13.2, and 4 kHz 72.7 ± 13.9; mean hearing level (dB) ±standard deviation in the control group: 0.25 kHz 60.6 ± 14.9, 0.5 kHz 67.5 ± 13.7, 1 kHz 63.1 ± 12.8, 2 kHz 70.6 ± 10.8, and 4 kHz 71.9 ± 10.0).

**Figure 1:**
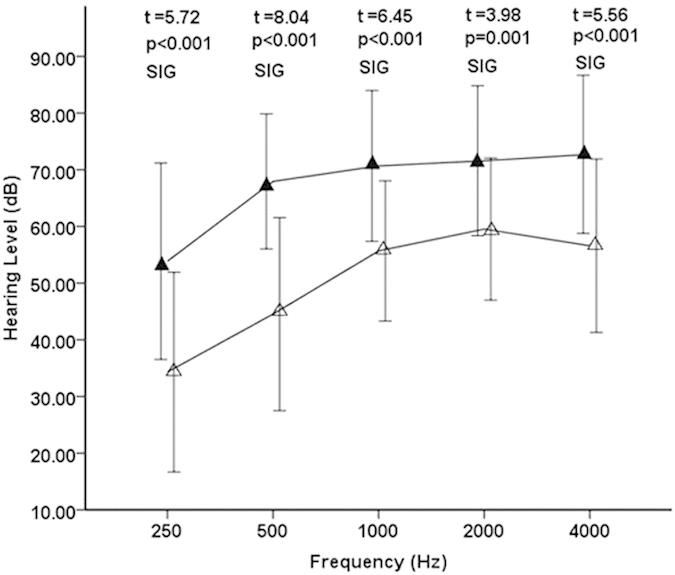
Initial and final hearing thresholds at 5 frequencies (250, 500, 1,000, 2,000, and 4,000 Hz) in the rTMS group. The filled and open triangles denote before and after rTMS treatment, respectively. Hz, Hertz; dB, decibel; t, Student’s t test; p, p value for significance of rejection of the null hypothesis; SIG, significant. The error bars denote the standard deviation.

**Figure 2:**
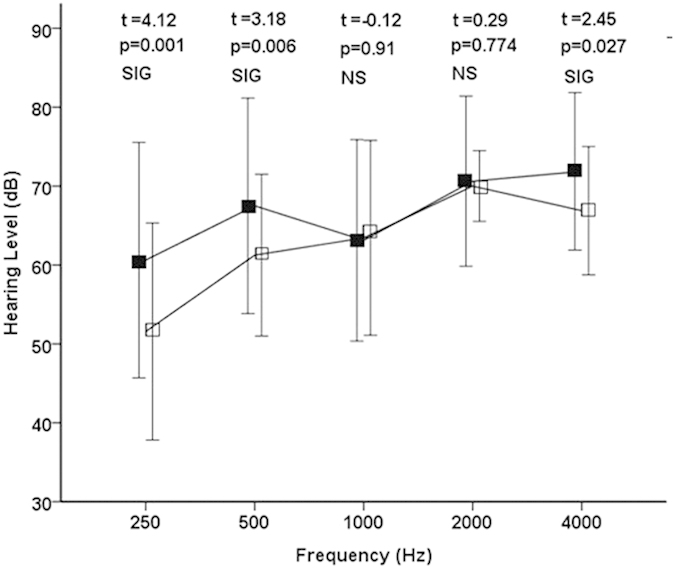
Initial and final hearing thresholds at 5 frequencies (250, 500, 1,000, 2,000, and 4,000 Hz) in the control group. The filled and open squares denote the mean hearing thresholds of before and after the secondary treatment, respectively. Hz, Hertz; dB, decibel; t, Student’s t test; p, p value for significance of rejection of the null hypothesis; SIG, significant; NS, non-significant. The error bars denote the standard deviation.

**Figure 3:**
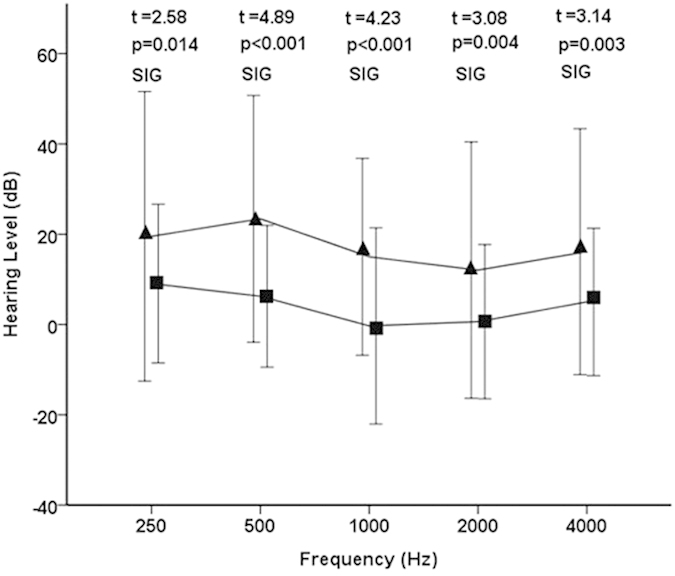
Hearing gain at 5 frequencies in the rTMS and control groups. Hz, Hertz; dB, decibel; t, Student’s t test; p, p value for significance of rejection of the null hypothesis; SIG, significant. The triangles and squares denote the mean hearing gains of the rTMS group and the control group, respectively. The error bars denote the standard deviation.

All patients tolerated rTMS without any serious side effects. The mean hearing thresholds at each of the 5 frequencies (0.25, 0.5, 1, 2, and 4 kHz) were calculated at the beginning and at the end of the rTMS treatment. Significant differences were detected in the thresholds at all frequencies between before and after rTMS treatment (Fig. 1; 0.25 kHz p < 0.001, 0.5 kHz p < 0.001, 1 kHz p < 0.001, 2 kHz p = 0.001, and 4 kHz p < 0.001). Although significant differences between the hearing threshold at 0.25 kHz, 0.5 kHz and 4 kHz were detected in the control group (Fig. 2; 0.25 kHz p = 0.001, 0.5 kHz p = 0.006, and 4 kHz p = 0.027), the hearing thresholds at 1 kHz and 2 kHz did not improve (1 kHz p = 0.91 and 4 kHz p = 0.774). The hearing gain in the rTMS group was significantly larger than that in the control group at all frequencies (Fig. 3; 0.25 kHz p = 0.014, 0.5 kHz p < 0.001, 1 kHz p < 0.001, 2 kHz p = 0.004, and 4 kHz p = 0.003).

**Figure 4:**
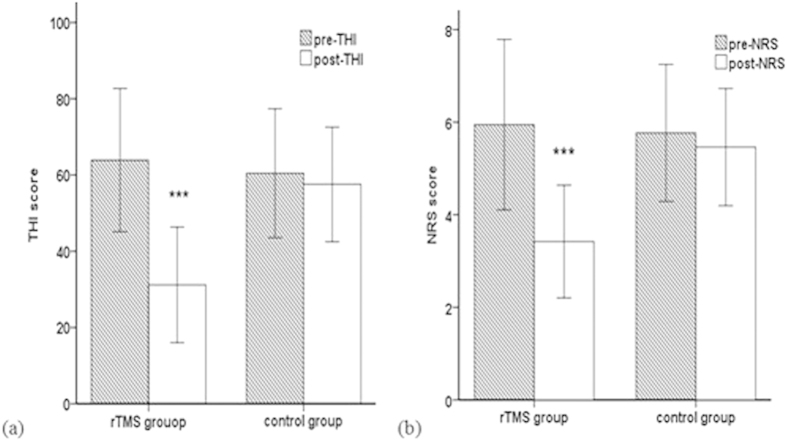
(**a**) Changes in the THI scores in the two groups of patients between pre- and post-secondary treatment. (**b**) The effects of rTMS on the patients’ estimated tinnitus volume based on the NRS scores pre- and post-secondary treatment. ***p < 0.001.

Nineteen patients from the rTMS group (55.9%; mean age 50.15 years, SD = 12.52) and 13 patients from the control group (65%; mean age 54.06 years, SD = 18.02) with tinnitus were analyzed for their tinnitus perception. Paired t-tests for dependent variables revealed a significant difference between pre- and post-rTMS in the Tinnitus Handicap Inventory (THI) score (Fig. 4a, t = 13.35, P < 0.001) and in the numeric rating scale (NRS) score (Fig. 4b, t = 9.04, P < 0.001). Our results showed a significant decrease between pre- and post-rTMS in the THI score (M = 63.89, SD = 18.79, and M = 31.16, SD = 15.16, respectively), and in the NRS score (M = 5.95, SD = 1.84, and M = 3.42, SD = 1.22, respectively). For the control group, a simple contrast analysis yielded no significant difference in the NRS score or the THI score between pre- and post-treatment. We analyzed that there was no linear correlation between hearing improvement and the changes noted in THI and tinnitus loudness NRS.

**Figure 5:**
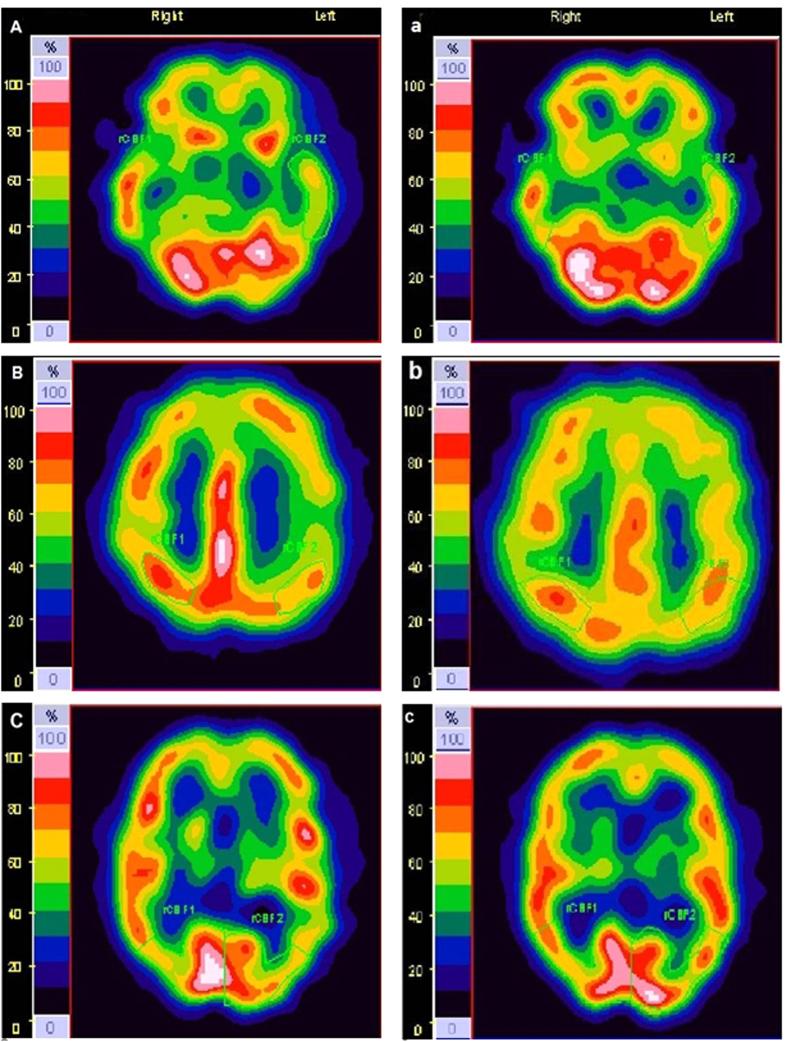
Representative SPECT images before rTMS ((A): temporal lobe; (B) parietal lobe; and (C): occipital lobe), and after rTMS (a–c), showing the areas that displayed a significant increase in rCBF in the patients with ISSHL after the application of low-frequency rTMS treatment over the TAC ipsilateral to the affected ear.

SPECT imaging showed that one patient with SSHL displayed decreased rCBF at multiple (4) foci; the other 4 patients displayed decreased rCBF at 1 focus. The comparison of rCBF between before and after rTMS based on SPECT imaging is presented in Table 1. Significant differences were observed in rCBF between pre- and post-rTMS (t = 6.015, P < 0.001). With the exception of the frontal lobe, the decrease in rCBF in the parietal, temporal, occipital and thalamus was alleviated to within 10% of the normal range after rTMS treatment. Representative SPECT images before and after rTMS treatment are shown in Fig. 5.

## Discussion

The present study demonstrates the favorable effect of rTMS treatment as a salvage therapy for sudden deafness. In the rTMS group, which received rTMS therapy in addition to the primary therapy, we observed a significant improvement in hearing thresholds compared to the control group, which received only SCT and HBO therapy. Because prior anatomical and PET imaging studies^23^,^24^ suggested that the temporoparietal junction (TPJ) (secondary and integrative auditory areas) is involved in tinnitus and because the application of rTMS to this area was previously successful^25^,^26^,^27^, we selected the TPJ as the target site. To the best of our knowledge, this is the first study to administer rTMS as a secondary therapy to patients who failed primary treatment with SCT and HBO therapy. We did not observe any apparent side effects of rTMS. Although allocation bias may have occurred in this historical cohort study, the more favorable outcomes of rTMS therapy compared with the control treatment provide some evidence supporting the beneficial effects of rTMS.

SSHL patients generally reach a fixed hearing level approximately 1 month after standard treatment^28^. All of the included subjects received SCT, which is currently the gold standard treatment, and HBO therapy, which is a well-accepted adjuvant therapy. The interval between the onset of SSHL and the beginning of rTMS for the patients in the rTMS group was longer than 1 month but shorter than 3 months. Therefore, the significant differences between the rTMS and control groups cannot be attributed to the primary therapy or a low recovery rate in the control group. In fact, these results reflect the good recovery rate in the rTMS group.

Another problem which should be considered is the hearing safety of rTMS in the experimental treatment of auditory disorders. The published data suggest that rTMS is a relatively safe and well-tolerated procedure for the treatment or diagnosis of pathologic positive sensory phenomena^29^. Moreover, rTMS improves auditory processing in healthy subjects and tinnitus patients^20^,^21^,^22^. Most studies have examined the effects of rTMS on the motor system using the motor-evoked potential (MEP) as a measure of cortical excitability; in general, low-frequency rTMS (≤1 Hz; ‘inhibitory’) has been shown to reduce MEPs, and vice versa for high-frequency rTMS (≥5 Hz; ‘excitatory’). 1-Hz rTMS applied to the auditory cortex cannot be regarded as generally inhibitory; its physiological effects depend on the preceding stimulation context, which is referred to as the state-dependency of rTMS^30^. Two studies have shown that no significant difference in hearing thresholds^31^,^32^ or in the presence of distortion-product otoacoustic emissions (DPOAE)^31^ was observed in healthy subjects exposed to one session of rTMS over the temporal cortex; for those subjects, who were protected by earplugs, the amplitude of transiently evoked otoacoustic emissions (TEOAE) decreased slightly within 1 hour after the rTMS session^32^. Based on an improvement in the PTA, our data support that rTMS represents a safe and effective therapy for ISSHL. To our knowledge, no study has reported the effects of rTMS on audiometry parameters in patients with ISSHL.

It has been clearly demonstrated that unilateral hearing loss can lead to auditory cortex reorganization^7^,^33^,^34^. Evidence of auditory plasticity in mature humans has been obtained from studying individuals with unilateral deafness^35^,^36^,^37^,^38^,^39^. In individuals with normal hearing, monaural stimulation results in asymmetrical activation of the central auditory system, in which the contralateral hemisphere produce greater activation than the ipsilateral hemisphere, which is referred to as “contralateral dominance”^40^. Based on EEG, MEG and fMRI studies, acute unilateral SSHL can induce functional reorganization in terms of altered hemispheric asymmetry for sound processing in the central auditory pathway in response to either affected- or healthy-ear stimulation^41^,^42^,^43^,^44^. In contrast to the pattern of “contralateral dominance” in control subjects with normal hearing, a pattern of “healthy-side dominance” was observed in ISSHL patients. Animal studies have revealed a down-regulation of both ipsilateral excitatory receptor expression/binding and contralateral inhibitory neurotransmitter synthesis with respect to the affected ear in the central auditory pathway; these studies demonstrated that the levels of excitatory and inhibitory neurotransmission-related proteins and neuroplasticity-related proteins in the auditory pathway were altered over time^45^,^46^. These findings suggest that these changes in protein expression may contribute to the molecular mechanism underlying the significant physiological changes in ISSHL patients, which lead to acoustic disorders following hearing loss. Li *et al.* demonstrated that a high degree of reorganization was associated with poor recovery from hearing loss in SSHL patients^35^. Thus, we performed rTMS as a new therapy based on the central auditory system remodeling theory.

One major limitation is that the underlying mechanisms that mediate the reported beneficial effects of rTMS are unclear. Several studies have aimed to determine whether rTMS disrupts the etiology of symptoms by altering activity in the cortex^47^,^48^,^49^. The multifocal brain perfusion abnormality may be due to the involvement of cognitive and emotional brain centers secondary to tinnitus and SSHL^50^. There is no clear explanation to justify why rTMS of the temporoparietal area resulted in increased rCBF in the temporal, parietal, and occipital lobes and the thalamus ipsilateral to the stimulation. Typically, rTMS for tinnitus is applied using a figure 8 coil. In this study, we used a circular coil, which is easier for locating the site and stimulates a more extensive area than the figure 8 coil. As a result, the rCBF of the temporal, parietal, and occipital lobes adjacent to the temporoparietal area may also be influenced. rTMS not only directly modulates superficial cortical areas but also indirectly affects remote areas that are functionally connected to the stimulated area, such as the auditory thalamus^51^. Along these lines, we observed an apparent increase in rCBF in the thalamus in patient 4 after rTMS treatment. The decrease in rCBF in the frontal lobe was alleviated to <10% in patients 1, 2, and 3, which may have been because the frontal lobe is distant from the stimulation site. We also observed that the hearing level and tinnitus did not remarkably improve in patient 2 or 3. The dorsolateral prefrontal cortex (DLPFC) is involved in auditory processing, exerts a bilateral facilitative effect on auditory memory storage and auditory attention, and contains auditory memory cells^52^, resulting in the top-down modulation of auditory processing^53^,^54^. Thus, the DLPFC is a promising stimulation site for further study^46^.

Although we cannot differentiate the extent to which these changes are related to rTMS or whether these changes reflect an improvement in the hearing level or a reduction in tinnitus severity, the decreased rCBF did elevated after rTMS. Consistent with previous studies, a significant increase in rCBF on the side ipsilateral to stimulation was observed during rTMS and persisted after discontinuation of stimulation in both humans and animals^55^,^56^,^57^. The exact mechanisms underlining the phenomenon have not been definitively delineated, though some study demonstrated that it was probably due to the autonomic components of the nerve, which dilated the cerebral arteries and increases cerebral blood flow when activated^55^.

A weakness of this study is that the outcome was measured 72 hours after TMS, without follow-up series to demonstrate a long lasting beneficial effect of rTMS. Additionally, we didn’t find a relationship between the improvement of hearing level and THI or tinnitus loudness NRS. Though the etiologies of tinnitus and SSHL are partially similar, such as fatigue and emotional stress, the pathogeneses are not the same. As a result, the severity of the two diseases is not synchronized, and the treatment mechanisms of rTMS for SSHL and tinnitus may be inconsistent as well. Another reason is probably that we lose statistical power with small sample sizes. Placebo-controlled studies examining a larger population and including long-term follow-up are required to better define the clinical efficacy and the treatment mechanisms of rTMS to relieve SSHL and tinnitus.

In conclusion, for SSHL patients exhibiting incomplete recovery following SCT and HBO therapy, the safety and feasibility of daily rTMS therapy over 4 weeks were demonstrated. These findings are the first to show the beneficial effects of rTMS on hearing outcomes over a 4-week period. Our findings are preliminary and suggest that the decreased rCBF was up-regulated after low-frequency rTMS procedure ceases, which need further study to define to what degree rTMS may contribute to it. In the present study, the participants autonomously decided whether or not to receive rTMS; thus, these results could theoretically be biased by motivational differences between the groups. We plan to conduct a randomized controlled multicenter trial to compare the effects of rTMS therapy alone to those of SCT. Compared to SCT, which can induce severe and potentially lethal side effects, such as infections, diabetes mellitus, and hypertension, rTMS therapy might constitute a safe and effective alternative for SSHL treatment.

## Materials and Methods

### Subjects

The study participants were recruited from patients seeking treatment at The First Affiliated Hospital of China Medical University who were diagnosed with SSHL. The Ethical Committee of the Institutional Review Board on Human Studies of our hospital approved this study, and the methods were carried out in accordance with the approved guidelines. All patients were required to sign an informed consent prior to participation in the study. We excluded SSHL cases with known etiology, including those whose hearing loss was caused by retrocochlear lesions, as detected by acoustic brainstem response and magnetic resonance imaging, and infectious or autoimmune diseases, as detected by laboratory examinations; thus, the diagnosis was considered to be idiopathic. The exclusion criteria also included bilateral SSHL, previous self or family history of SSHL, cardiac pacemaker or other implanted devices, intracranial metallic objects, neurological or psychiatric complications, pregnancy, and an inability to fulfill the study requirements. The PTA was calculated as the average of the thresholds at 5 frequencies (0.25, 0.5, 1, 2, and 4 kHz) at the beginning and at the end of the primary treatment; hearing gain of less than 10 dB at the end of the treatment was considered as treatment failure. Patients who were treated for longer than 4 weeks after the onset of sudden deafness were excluded from this study.

### Primary treatment

All SSHL patients enrolled in the study received systemic treatment for tinnitus at our hospital. These treatments included SCT (oral prednisolone 65 mg for 5 d, 30 mg for 2 d, 15 mg for 2 d, and 5 mg for 2 d). Then, the patients received 10 sessions of HBO therapy over a 2-week period. Patients who refused systemic treatment or received an initial steroid treatment at a different institute were excluded from this study.

### Secondary treatment

Patients who were refractory to systemic treatment were screened for entry into this study. The patients whose PTA did not improve by more than 10 dB between pre- and post-primary treatment were defined as refractory to systemic treatment. These patients were provided with 2 options for secondary treatment: rTMS therapy (rTMS group) and observation only, without any other treatment (control group). All patients were fully informed regarding the execution and goals of the study and provided written informed consent in accordance with procedures approved by the Ethics Committee of The First Affiliated Hospital of China Medical University.

The patients who selected rTMS therapy (rTMS group) received 1 Hz rTMS consisting of 1,200 stimuli per day for 20 sessions (Monday-Friday for 4 consecutive weeks) at 100% of their resting motor threshold (RMT). A circular, 125-mm diameter rTMS coil was utilized to administer rTMS (CCY-I, YIRUIDE, Wuhan, China). The stimulation site was over the temporoparietal association cortex (TAC) ipsilateral to the affected ear. The stimulation targets were defined using the 10–20 EEG electrode placement system. The surface marking of the stimulation point was centered over the left scalp between T3 and the midpoint of the line joining C3/T5; on the right scalp, the stimulation point was between T4 and the midpoint of the line joining T6/C4, in which the coil handle was directed backwards^58^. All rTMS therapy was performed by the same technician. During treatment, the coil was maintained in place using a mechanical arm.

The round coil was placed tangentially over the left primary motor cortex such that the handle pointed at a 45° angle posterolaterally. For the MEP measurement, surface electromyography (EMG) was recorded using pre-gelled, disposable Ag/AgCl electrodes by placing the active electrode in the right abductor brevis pollicis (thumb abductor), the reference electrode over the metacarpophalangeal joint, and the ground electrode over the wrist. The EMG signal was acquired at 3 kHz, which was filtered (10–500 Hz), amplified, and stored for offline analysis. The RMT was obtained over M1, which displayed TMS-induced MEPs of at least 50 μV at the lowest stimulus intensity in five out of 10 consecutive trials in the target muscle. All participants wore earplugs to protect them from possible acoustic trauma and to reduce noise from the discharge of the TMS coil.

### Patient evaluations

The PTA was calculated as the average of the hearing thresholds at 5 frequencies (0.25, 0.5, 1, 2, and 4 kHz) at the beginning (pre-PTA) and at the end (post-PTA) of the primary treatment. Additionally, the mean hearing threshold for each of the 5 frequencies (0.25, 0.5, 1, 2, and 4 kHz) was calculated for all patients at the beginning of the secondary treatment; this result was compared to that at 72 hours after the secondary treatment. The assessment of tinnitus loudness was performed using an NRS (range 0–10), whereas tinnitus severity was analyzed using the THI^59^.

### Neuroimaging

Neuronal activity was measured via SPECT before and within 1 week after rTMS. The patients were voluntary to conduct this examination. SPECT scans were performed on 5 patients from the active rTMS group, **who agreed to participate in SPECT measurement,** using a Starcam 3200i XR/T SPECT equipped with low-energy, high-resolution, parallel-hole collimators (GE, USA). The radiopharmaceutical Tc-99m ethyl cysteinate diethylester (^99m^Tc-ECD) was injected intravenously at a dose of 370 MBq 1 h after oral administration of 400 mg of calcium perchlorate and after 5–10 min of quiet rest without any sound or light stimulation (^99m^TcO4 was supplied by the Isotope Institute of China of the Atomic Energy Science Academy, and ECD was supplied by the Atomic Medical Institute of Jiang Su Province). The SPECT scan was performed immediately after ^99m^Tc-ECD was injected. A 555 MBq dose of ^99m^Tc-ECD was injected again 100 s after scanning. The data collection, imaging reconstruction and measurement of rCBF were performed according to previously reported methods^60^. Two experienced physicians from the Department of Nuclear Medicine confirmed the foci displaying decreased rCBF according to the reference criteria for an anomalous image of cerebral perfusion^61^. The rCBF of the foci was measured using an irregular region of interest. The mean rCBF of the foci and the respective contralateral (ml/100 g/min) site was recorded; the decrease in rCBF was calculated relative to that of the contralateral side. The difference between the 2 sides is typically less than 10%.

### Statistical analysis

SPSS software for Windows (version 20.0; SPSS Inc., Chicago, IL, USA) was used for analysis. We calculated the mean hearing threshold differences across all frequencies between ears to estimate the degree of hearing recovery due to rTMS treatment. The data were compared between the rTMS and control groups using the t test. Parametric paired t-tests were used for within-group comparisons of dependent variables. The data in the text are presented as the means ± standard deviation. A p value < 0.05 was considered to be statistically significant. All statistical tests were 2-sided.

## Additional Information

**How to cite this article**: Zhang, D. and Ma, Y. Repetitive transcranial magnetic stimulation improves both hearing function and tinnitus perception in sudden sensorineural hearing loss patients. *Sci. Rep.* **5**, 14796; doi: 10.1038/srep14796 (2015).

## Figures and Tables

**Table 1:** The comparison of rCBF between before and after rTMS based on SPECT imaging.

Patient number(affected side)	Foci based onSPECT	rCBF of the foci(ml·100 g^−1^·min^−1^)		Extent of the decrease inrCBF (%)	
		Pre-rTMS	Post-rTMS	Pre-rTMS	Post-rTMS
1 (Left)	Frontal lobe				
	Left	55.45	53.87	15.11%	12.83%
	Right	65.32	61.80		
	Parietal lobe				
	Left	54.91	62.82	14.24%	5.92%
	Right	64.03	59.10		
	Temporal lobe				
	Left	51.08	59.24	15.60%	0.40%
	Right	60.52	59.48		
	Occipital lobe				
	Left	65.48	68.09	12.48%	3.95%
	Right	74.82	70.89		
2 (Left)	Frontal lobe				
	Left	51.53	54.75	16.58%	10.14%
	Right	61.77	60.93		
3 (Left)	Frontal lobe				
	Left	52.12	53.01	16.95%	12.21%
	Right	62.76	60.38		
4 (Left)	Thalamus				
	Left	64.52	69.05	13.00%	4.26%
	Right	74.16	72.12		
5 (Right)	Parietal lobe				
	Left	57.89	59.78	13.07%	2.14%
	Right	66.59	61.09		
